# Reappraising the role of thyroid scintigraphy in the era of TIRADS: A clinically-oriented viewpoint

**DOI:** 10.1007/s12020-024-03825-0

**Published:** 2024-04-16

**Authors:** Pierpaolo Trimboli, Joerg Bojunga, Maurilio Deandrea, Francesco Frasca, Alessio Imperiale, Andrea Leoncini, Gaetano Paone, Fabian Pitoia, Mario Rotondi, Ramin Sadeghi, Lorenzo Scappaticcio, Giorgio Treglia, Arnoldo Piccardo

**Affiliations:** 1 Servizio di Endocrinologia e Diabetologia, Ente Ospedaliero Cantonale (EOC), Lugano, Switzerland; 2 Facoltà di Scienze Biomediche, Università della Svizzera Italiana (USI), Lugano, Switzerland; 3 Department of Medicine I, Goethe University Hospital, Theodor-Stern-Kai 7, Frankfurt am Main, Germany; 4 Endocrinology, Diabetes and Metabolism Department and Center for Thyroid Diseases, Ordine Mauriziano Hospital, Turin, Italy; 5 Endocrinology Section, Department of Clinical and Experimental Medicine, Garibaldi Nesima Hospital, University of Catania, Catania, Italy; 6 Nuclear Medicine and Molecular Imaging, Institut de Cancérologie de Strasbourg Europe (ICANS), Strasbourg University Hospitals, Strasbourg, France; 7 Molecular Imaging, DRHIM, Institut Pluridisciplinaire Hubert Curien (IPHC), UMR7178, CNRS, University of Strasbourg, Strasbourg, France; 8 Servizio di Radiologia e Radiologia Interventistica, Istituto di Imaging Della Svizzera Italiana (IIMSI), Ente Ospedaliero Cantonale (EOC), Lugano, Switzerland; 9 Clinic of Nuclear Medicine, Imaging Institute of Southern Switzerland, Ente Ospedaliero Cantonale (EOC), Bellinzona, Switzerland; 10 Head Thyroid Section, Division of Endocrinology, Hospital de Clinicas, University of Buenos Aires, Viamonte, Argentina; 11 Department of Internal Medicine and Therapeutics, University of Pavia, Pavia, Italy; 12 Unit of Endocrinology and Metabolism, Laboratory for Endocrine Disruptors, Istituti Clinici Scientifici Maugeri IRCCS, Pavia, Italy; 13 Nuclear Medicine Research Center, Mashhad University of Medical Sciences, Mashhad, Iran; 14 Unit of Endocrinology and Metabolic Diseases, AOU University of Campania “Luigi Vanvitelli”, Naples, Italy; 15 Department of Advanced Medical and Surgical Sciences, University of Campania “Luigi Vanvitelli”, Naples, Italy; 16 Faculty of Biology and Medicine, University of Lausanne, Lausanne, Switzerland; 17 Department of Nuclear Medicine, E.O. “Ospedali Galliera”, Genoa, Italy

## Abstract

Thyroid nodules (TNs) are a common entity, with the majority being benign. Therefore, employing an accurate rule-out strategy in clinical practice is essential. In the thyroid field, the current era is significantly marked by the worldwide diffusion of ultrasound (US)-based malignancy risk stratification systems of TN, usually reported as Thyroid Imaging Reporting And Data System (TIRADS). With the advent of US (and later TIRADS), the role of thyroid scintigraphy (TS) in clinical practice has gradually diminished. The authors of the present paper believe that the role of TS should be reappraised, also considering its essential role in detecting autonomously functioning thyroid nodules and its limited contribution to detecting thyroid cancers. Thus, this document aims to furnish endocrinologists, radiologists, surgeons, and nuclear medicine physicians with practical information to appropriately use TS.

## Background

The thyroid nodule (TN) is a common pathological entity occurring in up to 60–70% of adults [1]. The majority of TNs are benign, with a cancer prevalence per patient estimated lower than 2% [2]. The huge number of benign TNs encompasses hyperplasia, several types of adenomas, and inflammatory lesions within autoimmune thyroid disorders [3]. Among thyroid malignancies there are differentiated carcinomas, medullary carcinoma, and other rare cancers such as undifferentiated/anaplastic, lymphoma, and metastases from other organs [3]. Thus, using an accurate rule-out strategy in clinical practice is essential. In the thyroid field, the current era is marked by the worldwide diffusion of ultrasound (US)-based malignancy risk stratification systems of TN, developed by most important international societies and usually reported as Thyroid Imaging Reporting And Data System (TIRADS) [4–6]. These systems were conceived to standardize the lexicon of thyroid US and reduce as much as possible the indication for fine needle aspiration cytology (FNAC). Indeed, the literature has confirmed that TIRADSs are reliable in detecting thyroid cancer, and papillary carcinoma (PTC) in particular, and saving on unnecessary FNACs (i.e., biopsies performed with benign cytological report). Regarding the latter, some discrepancies have been observed among TIRADSs, being the rate of unnecessary FNAC lower according to ACR-TIRADS as compared to the other ones. However, evidence-based studies have primarily tested TIRADS performance against PTC [7], while the other cancers and benign lesions may present differently at US with consequent heterogeneous assessment according to TIRADS. An important project endorsed by international societies is currently ongoing to develop the international TIRADS which should solve the weaknesses of the TIRADSs and will replace them in the future [8]. Integrating patients’ clinical features and results of other imaging procedures into the interpretation of TIRADS assessments should be optimal for clinicians.

Among the other thyroid imaging procedures, thyroid scintigraphy (TS) was traditionally used as the initial TN evaluation until 1990’s when US become pivotal in this diagnostic work-up. With the advent of US (and later TIRADS), the role of TS in clinical practice was progressively debunked, and its indication is currently limited to some specific conditions [9]. However, some benign pathological entities of TN cannot be identified by TIRADS, and TS may contribute to improving the performance of TIRADSs in reducing the rate of unnecessary FNACs. In addition, patients referred with inconclusive cytological report may be addressed to treatment alternative to surgery depending on the TS pattern. From the functional point of view, while thyroid cancer generally presents with low or absent uptake at TS, functioning nodules can be encountered among benign lesions; in particular, autonomously functioning thyroid nodule (AFTN) is the term conventionally used to define those TNs that overproduce hormones as to exceed the body tissues requirement. In the context of the need to improve the performance of US and TIRADSs, whether we should exclude AFTNs before FNAC with the aim of further reducing unnecessary FNACs is currently a matter of debate. AFTNs are virtually benign but have heterogeneous US presentation and often large size, being non negligible the likelihood to indicate (unnecessary) FNAC in these nodules [10–12]. However, anecdotal cases of hyperfunctioning/toxic cancers have been described in the literature [13, 14]. Furthermore, considering that TIRADS was based on the US presentation of PTC and can overlook follicular and medullary carcinoma, whether TS can still hold a role in cancer risk stratification should be re-discussed. Thus, a reappraisal of the role of TS in the era of TIRADS diffusion is needed to furnish endocrinologists, radiologists, surgeons, and nuclear medicine physicians with practical information.

## Recommendations about thyroid scintigraphy from international guidelines

As the number of subjects with TNs is substantial, a well-defined diagnostic work-up has been worldwide implemented. Guidelines in this field have been published by international societies providing clinicians with recommendations for appropriate assessment of TN [15, 16]. Despite some marginal specific issues, guidelines substantially agree about the work-up to follow. Briefly, physical examination remains useful to select patients requiring imaging evaluation, testing for TSH (with or without free-T3 and free-T4) is needed to assess thyroid function, US is recognized as the pivotal imaging procedure to assess the risk of malignancy of any TN, TS by using sodium-iodide symporter (NIS) targeting tracers (TSn), such as ^123^I natrium (sodium) iodide (^123^I) or ^99m^Tc-natrium (sodium) pertechnetate (^99m^TcO_4_^-^), is indicated when AFTN-related (clinical or subclinical) hyperthyroidism is suspected [17]. In addition, while TS with proliferation target tracer (^99m^Tc-sestamibi [TSp]) has been proposed as potential tool able to better estimate the risk of cancer by nuclear medicine guidelines [17], neither ETA nor ATA TN guidelines provide any specific clinical recommendation about it [15, 16].

## Purpose of the paper

The authors of this paper critically reappraised the issue of indication for TS in thyroid nodule(s) adult patients in the current era of TIRADS-oriented management of TN patients. This document has to be intended as an easy-to-use guide to furnish endocrinologists, radiologists, surgeons, and nuclear medicine physicians with practical information to appropriately use TS, also considering the implications associated with choices.

## Essentials

1. Thyroid ultrasound
  - US is recognized as the most accurate imaging procedure to estimate the thyroid size and evaluate thyroid morphology and structure.
  - US is recognized as the most accurate imaging tool to detect TNs, estimate their size, and stratify their risk of malignancy.
  - TIRADSs are universally recognized as reliable to assess the risk of malignancy of TNs.
  - FNAC is worldwide indicated according to TIRADS assessment, size of the lesion, and eventual individual risk factors.
2. Thyroid scintigraphy
  - Scintigraphy is the imaging procedure providing information about the function of TNs.
  - According to TSn uptake, TNs are classified as hypo-, iso-, and hyperfunctioning, being the latter pattern considered as virtually benign.
  - The choice of treatment of AFTN significantly depends on TSn pattern.
3. Combining thyroid ultrasound and scintigraphic data
  - Performing and interpreting thyroid US is generally not influenced by scintigraphic data.
  - Data of the thyroid morphology at US is essential for the optimal interpretation of TS.
  - Thyroid US should be always performed before TS, and nuclear medicine physician should be aware of thyroid US data and specific indication for TS.

## Advantages and implications of performing TSn to detect AFTNs

### Scope of the problem

The prevalence of AFTN varies depending on the population and geographical location. While the prevalence of TN in the general population is estimated up to 70%, the occurrence of AFTN is lower, between 2.7% and 4.4% [18]. In regions still transitioning from iodine-deficient to iodine-sufficient areas (such as parts of South America and Europe), AFTN can account for a higher proportion of cases [19]. AFTN is generally single, multiple AFTNs being rarer. AFTN is usually recognized as benign entity, with very rare exception of malignant carcinoma with high uptake at TSn. The term AFTN encompasses various pathological entities, including adenoma and hyperplasia [20].

AFTNs may present in euthyroid young subjects and, theoretically, they can progress over time with increase in size and, as direct consequence, in hormonal production. The timing of this progression is unknown. However, AFTNs with clinical impact are mostly observed in elderly, with some exceptions in young adults. Essentially, AFTN can have a clinical impact only when it is toxic. Hyperthyroid patients with toxic AFTN can significantly benefit from treatment.

### Indication for treating AFTN patients and available options

The options of treatment of AFTN patients traditionally include radioiodine (RAI) and surgery. Ideally, RAI represents the preferred option due to its high efficacy and quite absent related side-effects. In many countries, RAI is performed with about five-day hospitalization, while in other regions patients are discharged just after the RAI administration. Hemithyroidectomy is the primary and effective alternative to RAI. This operation may take about one hour and needs one day of hospitalization. While the resolution of AFTN-related hyperthyroidism after surgery is immediate, patients undergoing RAI can achieve euthyroidism after some months. In addition, RAI may be ineffective or sometime induce permanent hypothyroidism. More recently, thermal ablation has shown promising results, although long-term data are still not available [21]. Thermal ablation, or surgery, can be preferred in the setting of symptomatic patients having AFTN non-suitable for RAI. Finally, elderly individuals with significant comorbidities and contraindications to surgery may be treated with long-term therapy using oral anti-thyroid medications (i.e., thionamides) [22, 23].

Significantly, finding AFTNs in euthyroid patients has not direct therapeutic consequence. These patients are usually asymptomatic and are addressed to long-term clinical follow-up. Radioiodine is not clinically required in these cases due to the considerable risk to ablate normal thyroid parenchyma and then determine hypothyroidism.

### Implications of non-considering TS before FNAC

Studies have raised the question of whether AFTN should be excluded before indicating FNAC [10–12]. This is based on the fact that performing FNAC in AFTN may lead to indeterminate report (i.e., Bethesda III or IV) with consequent difficult management and “risk” of unnecessary diagnostic surgery in patient with virtually benign lesion. Clinicians may wonder about the implications of recommending diagnostic surgery for a patient with AFTN. Firstly, the cancer prevalence in patients with toxic nodular goiter is estimated up to 9% which is comparable to that of multinodular goiter in general [24]. Secondly, when TSn is routinely performed, the prevalence of AFTN among nodules with FNAC indication was low as up to 7.6% [25]. Overall, the rate of AFTNs assessed at high risk according to TIRADSs is below 5% [10–12, 25]. Thirdly, careful interpretation of cytological indeterminate samples is mandatory also in AFTN identified on TSn. Fourthly, as AFTNs have heterogeneous US presentation, and considering that the presence of cancer in a “hot” nodule cannot be excluded a priori, FNAC can be performed in AFTNs without harm, especially when they are at high risk according to TIRADS. Fifthly, the data about the issue of indeterminate FNAC report in AFTNs are sparse [12]. This can be associated with autonomous/toxic adenomas [20]. Sixthly, the possibility of faint uptake on TSn with ^99m^TcO_4_^-^ should be considered because trapping-only nodules occur with up to 5% prevalence [26]. Seventhly, surgery represents in any case an option of treatment of AFTN [22], as well as the indeterminate TNs, and hemithyroidectomy generally does not determine hypothyroidism and it is associated with very low risk of complications. In this context, hyperthyroidism guidelines are strongly focused on the pre-RAI preparation and recommend to exclude cancer before RAI [22]. Finally, AFTN has to be considered among ^18^F FDG PET/CT incidentalomas [27]; this pattern can harbor hyperfunctioning adenomas with low TSH [20] thus meaning that clinical and laboratory assessment is needed before indicating FNAC in ^18^F FDG PET/CT incidentalomas.

### Clinically-oriented remarks

The worldwide experience of the last two/three decades is that the number of patients with indeterminate FNAC report undergoing diagnostic surgery is too high. In fact, the largest part of patients has benign histology. Subsequently, significant efforts have been made in guidelines and clinical practice to minimize as much as possible interventions. In this regard, since TIRADSs have demonstrated high performance for risk stratification of TNs, a TIRADS-based work-up remains the preferred approach. Since TSn is the gold standard for detecting AFTN, it has to be considered in patients clinically suspected as harboring toxic AFTN (i.e., theoretically in presence of TSH suppressed or at least below the lower reference limit). According to the literature, the “risk” to perform inappropriate FNAC in AFTNs from consecutive series of TN patients is negligible. Basically, the strategy including TSn to reduce unnecessary FNAC is not economically sustainable, and clinicians are asked to manage TN patients according to their individual characteristics as well as thyroid tests, and TSn when indicated. Additionally, TSn may be considered in patients with indeterminate FNAC report and clinical/US clues for (mild) hyperthyroidism. Finding uptake at TSn in TN with indeterminate FNAC report may save on an unnecessary surgery, even if the possibility of cancer must be discussed with patient and in multidisciplinary context. Even if no high-quality data are available in this regard, it looks like reasonable consider TSn in patients with indeterminate FNAC, especially in elderly cases who are at higher risk to harbor AFTN. According to the literature, there is no role of TSp. In any case, TIRADS assessment of TN should guide the choice of treatment.

## Advantages and implications of performing thyroid scintigraphy to detect cancers

### Scope of the problem

Non-hyperfunctioning lesions, known as “cold nodules” on TSn represent the vast majority (up to 90%) of all TNs identified with US. Consequently, the low or absent uptake on TSn does not correlate with the risk of malignancy. Indeed, even very low-risk thyroid lesions, such as predominantly cystic ones, often exhibit minimal or absent uptake on TSn. From this point of view, TSn should be never considered to evaluate the risk of malignancy of the nodules and their indication for FNAC.

### Implications of finding cold TN

A cold TN at TSn does not definitively indicate malignancy. When TSn is used to assess the risk of malignancy, cancer is detected in only up to 5.3% of cases [28] or even significantly lower (1.1%) when looking at the level of primary/secondary care with a significant lower pre-test probability of having cancer [2]. Then, further assessment of cold TN, firstly by US with or without FNAC, remains essential. Due to the well-recognized limitations of TSn in detecting thyroid cancer, TSp has been proposed over the past 25 years as a complementary test to TSn to improve the stratification of malignancy, particularly in TNs with indetermined cytology. Although a high negative predictive value of TSp was reported for ruling out differentiated carcinoma, studies remain limited [29], and evidence-based data found suboptimal sensitivity and specificity of TSp for discriminating malignant from benign TNs, thus suggesting that TSp cannot be useful in clinical practice [30]. In the current era marked by TIRADSs, molecular tests, and the updated and significantly improved classifications of thyroid cytology, many doubts about the role of TSp remain. Indeed, other molecular imaging procedures such as ^18^F-FDG PET/CT [31–33], has gained momentum in the field of nuclear thyroidology. Evidence-based data supports using ^18^F-FDG PET/CT in highly selected scenarios [34, 35]. Also, ^18^F FDG PET/CT showed higher performance as compared to that of TSp in the setting of TN with indeterminate FNAC [32].

### Clinically-oriented remarks

The use of TSn, either alone or in combination with TSp, does not aid in stratifying the risk of malignancy of TNs and cannot improve the performance of TIRADSs. Its use should be limited, according to evidence-based studies and in line with Choosing Wisely campaign of the Society of Nuclear Medicine and Molecular Imaging (SNMMI) [36].

## Conclusions for clinical practice

The current era of medicine is significantly marked by the need of limiting as much as possible unnecessary diagnostic tests and their related costs. In the thyroid field, clinicians and US operators are asked to save on unnecessary FNACs with the intent to reduce, and ideally avoid, further inconvenient implications. Therefore, thyroidologists should also aim to avoid other unnecessary diagnostic tests and imaging procedures, especially the low cost-effective ones. Over the last two decades, thyroid US has achieved such a high clinical performance that TN patient management is primarily guided by US/TIRADS. As a result, the role of TS has significantly evolved. Indeed, its use to stratify the risk of malignancy is substantially discharged and no longer recommended by guidelines. Even if TSn remains the gold standard for detecting AFTN, considering the high reliability of TIRADS in risk stratification of TNs and the possibility to find a cancer in toxic TN, TSn can be appropriately recommended before FNAC in patients with suspected solitary toxic AFTN (i.e., low/suppressed TSH), also to determine their eligibility for RAI. In addition, TSn can be useful to detect both toxic and non-toxic AFTN among TNs with indeterminate FNAC, also in the attempt to reduce the resection rate of these cases. Anyway, FNAC can be performed according to TIRADS without harms, ad TSn can be used as second-line imaging, when clinically appropriate. In any case, FNAC must be performed in TNs assessed as high-risk according to TIRADS, independently of their TS pattern.
